# The unidentified hormonal defense against weight gain

**DOI:** 10.1371/journal.pbio.3000629

**Published:** 2020-02-25

**Authors:** Jens Lund, Camilla Lund, Thomas Morville, Christoffer Clemmensen

**Affiliations:** Novo Nordisk Foundation Center for Basic Metabolic Research, Faculty of Health and Medical Sciences, University of Copenhagen, Copenhagen, Denmark

## Abstract

Human biology has evolved to keep body fat within a range that supports survival. During the last 25 years, obesity biologists have uncovered key aspects of physiology that prevent fat mass from becoming too low. In contrast, the mechanisms that counteract excessive adipose expansion are largely unknown. Evidence dating back to the 1950s suggests the existence of a blood-borne molecule that defends against weight gain. In this article, we discuss the research supporting an “unidentified factor of overfeeding” and models that explain its role in body weight control. If it exists, revealing the identity of this factor could end a long-lasting enigma of energy balance regulation and facilitate a much-needed breakthrough in the pharmacological treatment of obesity.

## Abstract 2

The endocrine signals governing the homeostatic regulation of body weight are incompletely mapped; this Unsolved Mystery article discusses the mounting evidence that points to the existence of unidentified hormonal factors protecting against excessive weight gain.

## Obesity and its biological roots

**Fig 1:**
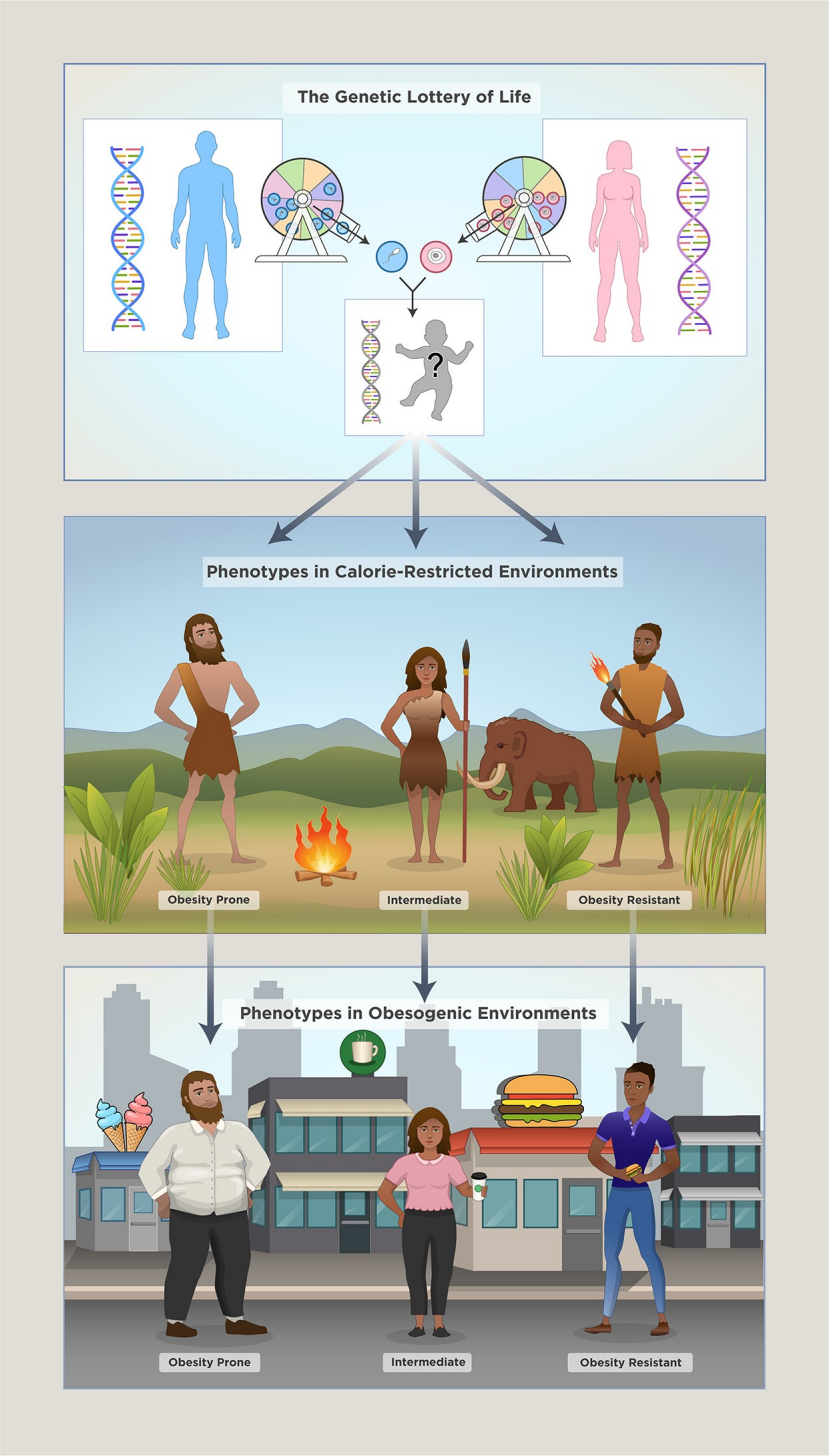
Body fat phenotypes are determined by the “genetic lottery of life” and socioenvironmental factors. A complex interplay between random genetic and epigenetic factors and social and environmental factors determines human fatness. In restrictive environments, i.e., environments where high energy expenditure is required to obtain few calorie-poor foods, variation in body weight is low simply because a subchronic negative energy balance prevents obesity-prone individuals from putting on weight. Conversely, obesogenic environments are characterized by high availability of hyperpalatable foods, and minimal physical efforts are required to obtain the next calorie-dense meal. Such environments reveal (1) parts of the population that are genetically predisposed to obesity (obesity prone), (2) individuals that only put on a moderate amount of fat mass (intermediate), and (3) individuals who have inherited a genetic “advantage” that allows them to stay lean (obesity resistant).

Historically, fatness was a desirable attribute associated with social status, wealth, and fertility [1,2]. Although this view is still dominant in certain cultures, the Western world recognizes adiposity as a chronic condition that hampers human health [3]. Individuals with obesity are often stigmatized [4], and many lean individuals erroneously believe that severe overweight is a self-inflicted situation caused by eating too much and exercising too little. The simplicity of this thinking is opposed by the complex causes of obesity [5] and by the “brainteasing” biology that makes it very hard for millions of people to fight their own fat mass [3]. Biomedical assistance is likely needed to win this battle, and in order to provide this support, perhaps it is time for obesity scientists to consider the less-beaten research paths. Instead of searching for yet another slimming agent, it might be better to reveal why some people easily put on pounds while others stay lean. In contrast to conventional assumptions about superior self-control and willpower, human studies have demonstrated that weight gain resistance has deep biological roots [6,7] (**Fig 1**). Characterizing these is among the critical steps toward an improved understanding of obesity etiology. Importantly, geneticists have finally started to uncover the genome of thinness [8,9], but their efforts might be fruitless unless physiologists determine the fundamental features of fat mass regulation encoded by these genes.

## Between biological boundaries: How do mammals maintain a stable body weight?

Despite wide variations in day-to-day food intake and physical activity, body weight remains rather stable throughout adulthood [10]. If one considers how many tons of food we ingest during midlife, the approximately 10 kg often gained during this time span represents an energetic “error” of about 0.2% [11]. This remarkable accuracy indicates that body weight is under autonomous regulation, a notion that is supported by animal studies. For example, when rats are fed energy-diluted diets or are treated in ways that elevate their metabolic rate, they respond by increasing food intake to an extent that defends their normal body weight [12]. Conversely, when forced into a positive energy balance by infusion of liquid calories, either by gavage or via implanted gastric tubes, animals compensate by lowering their voluntary intake of food [13–17]. Moreover, voluntary feeding completely stops if the quantity of infused calories is sufficiently high. What is even more striking is that once forced overfeeding ceases, hypophagia continues until body weight has returned to baseline [13,14]. The potency of this response is illustrated by 2 rhesus monkeys that responded to prolonged overfeeding by not ingesting any foods for up to 35 days [13].

These findings strongly suggest that overall energy balance is achieved by a homeostatic feedback system that matches energy intake with expenditure. Research into this aspect of mammalian physiology took off in the middle of the 20th century [18], and lesion studies in rodents quickly highlighted the hypothalamus as a crucial component of this system [19]. This work was followed by a series of “parabiosis” studies in which rats were surgically connected to one another, creating a shared circulation [20]. These experiments not only provided evidence for the existence of a circulating satiety signal but also favored Kennedy's lipostatic theory [21] that a blood-borne signal from fat tissue informs the brain of the size of adipose stores [22,23]. Identifying this factor, however, turned out to be a rather challenging task, and initial biochemical purifications of adipose extracts did not provide much insight [24]. Instead, it was the emerging molecular biological era of the late 1980s that provided the first clue that a specific transcript was preferentially induced in adipose tissue of overfed animals [14]; later, this work culminated with the identification of leptin as an endocrine master-regulator of body weight homeostasis [25,26].

The landmark discovery of leptin accelerated research into the neuroendocrine basis of food intake. At the turn of the 21st century, an influential model depicted leptin as a long-term satiety signal that controls energy intake in concert with short-acting hormones released from the gut upon ingestion of meals [27], and very soon thereafter, ghrelin was reported to be a key blood-borne hunger signal protecting against a negative energy balance [28]. This conceptual model is in line with the popular set-point theory, which states that body weight is under tight biological regulation and that any change in adipose mass will be compensated for by adjustments in energy intake and expenditure, causing a rapid return to the “set” level of adiposity [29]. If such a strong regulatory system really exists, fat mass should not be affected by various life events such as starting college or getting married. But circumstances like these do alter adiposity in many cases, highlighting a major limitation of this model [29].

**Fig 2:**
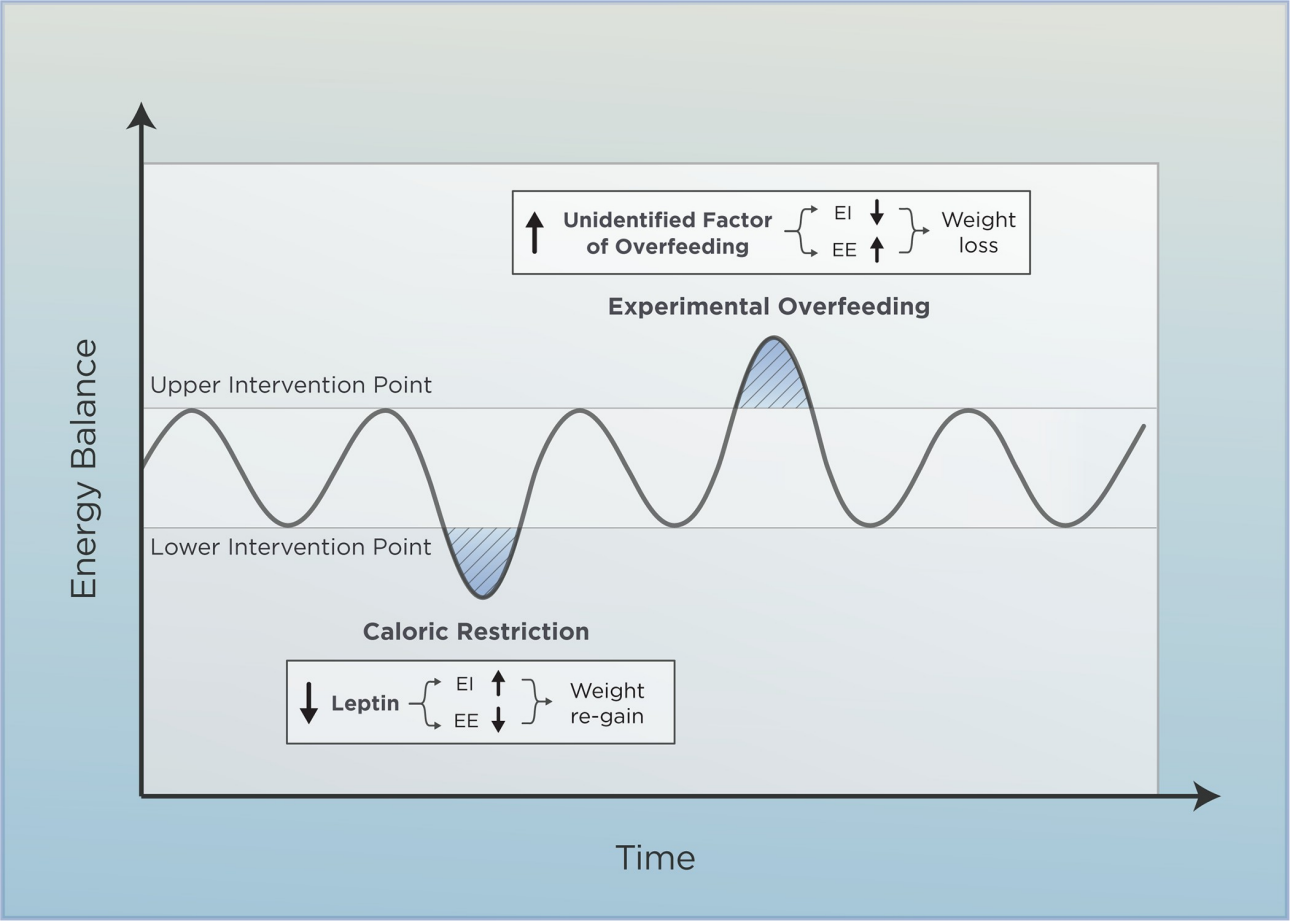
Homeostatic regulation of body weight. This simplified model suggests that homeostatic mechanisms protect against weight perturbations in either direction. The adipose-derived hormone leptin plays a key role in maintaining the “lower intervention” point in response to caloric restriction. Conversely, an “unidentified factor of overfeeding” has been hypothesized to counteract the overfed state around the upper intervention point. For already well-described factors implicated in body weight regulation, see references [7,34]. EE, energy expenditure; EI, energy intake.

The dual-intervention point model accounts for the fact that fatness appears to be both under biological control and influenced by social factors [29,30]. As its name implies, this model argues that body weight is constrained by 2 biological boundaries rather than being tweaked around a specific set point. In between these boundaries, i.e., the upper and lower point of intervention, there is a “zone of biological indifference” in which socioenvironmental factors predominantly affect fat mass. Because physiological regulators are less active within this zone, fat mass can fluctuate freely until it “bounces into” either the upper or lower boundary. When this happens, powerful biological feedback forces are engaged to ensure that adipose depot size does not change to an extent that jeopardizes the ability to escape from life-threating dangers or to survive periods of starvation and sickness-induced anorexia [31]. Solid evidence supports that low circulating leptin acts as a strong starvation signal by potentiating appetite and protecting against a dangerous degree of thinness [7,32,33]. Consequently, leptin is considered an important mediator of the lower intervention point [31] (**Fig 2**). Although research into this aspect of body weight regulation has revealed one explanation why it is so difficult to maintain a large weight loss [3], it provides no clue as to why some individuals become obese in the first place. To answer this crucial question, we have to investigate the upper intervention point and its underlying biology.

## Why are we not all obese? Evolutionary and environmental perspectives on weight gain propensity

It is estimated that overweight affects more than 2 billion people worldwide [35]. This number may not come as a surprise, given the global emergence of fattening food environments [36]. Yet, if the vast majority of the global population is exposed to strong obesogenic stimuli, why is it that only approximately 600 million people are obese? The observation that some individuals remain remarkably lean throughout life conflicts with the popular “thrifty gene” hypothesis, an idea that proposes that famines have been common in human history, and adiposity-promoting alleles within the genome have been subjected to strong positive selection during such devastating events. If this hypothesis is true, it has been argued that evolution should have shaped the genetic makeup of mankind in a manner that would make all modern humans obese [37]. As this is clearly not the case, something else must explain why only a subset of the population is prone to weight gain.

In order to understand what that might be, it may help to take a closer look at the opposite phenomenon, weight gain resistance. Extreme examples of this can be seen in persons who are naturally thin and nonanorectic. This phenotype has been termed persistent thinness [38] or constitutional thinness [39] and is defined by a body mass index of ≤17.5 kg/m^2^ but being otherwise healthy. One intriguing characteristic of these individuals is their desire to gain weight and their self-reported difficulties in doing so [39,40]. Interpreting this phenotype in light of the dual-intervention point model, it can be rationalized that the upper intervention point in constitutionally lean subjects must be under narrow biological control and that the homeostatic defense mechanisms against fatness are engaged even upon very small increases in adipose mass. In contrast, people who are prone to weight gain might be so because their upper intervention point is located at a markedly higher level. Their zone of biological indifference is correspondingly larger, and the defense systems that are supposed to prevent weight gain only become activated upon extensive adipose tissue expansion. In an obesogenic environment, their fatness is therefore pushed toward their relatively higher upper biological boundary.

But why is it that we seemingly differ so much when it comes to weight gain propensity? A growing line of evidence suggest that the modern food environment promotes hedonic overeating. Anticipated pleasure associated with eating a palatable diet combined with an increased availability of highly rewarding foods drives susceptible individuals to eat in the presence of metabolic satiety [41]. According to another idea, the drifty gene hypothesis [37], there could also be an evolutionary explanation for why some individuals easily gain weight. Early hominids harboring a less sensitive or defective fat mass defense system would have been wiped out by natural selection because of their increased predation risk. However, once our ancestors refined social behavior and collaboration and invented fire and weaponry, they rose to the top of the food chain, suggesting that the evolutionary forces that used to limit adiposity were suddenly no longer favored. Because of the subsequent lack of selection pressure, the encodings of the upper intervention point became subject to erosion by random mutations, and as a result, we now differ widely in terms of defending ourselves against a prolonged excessive energy intake [29–31,37]. This differential response is clearly evident in both natural experiments and in controlled human overfeeding interventions.

## A damaged defense: How do modern humans respond to overfeeding?

**Fig 3:**
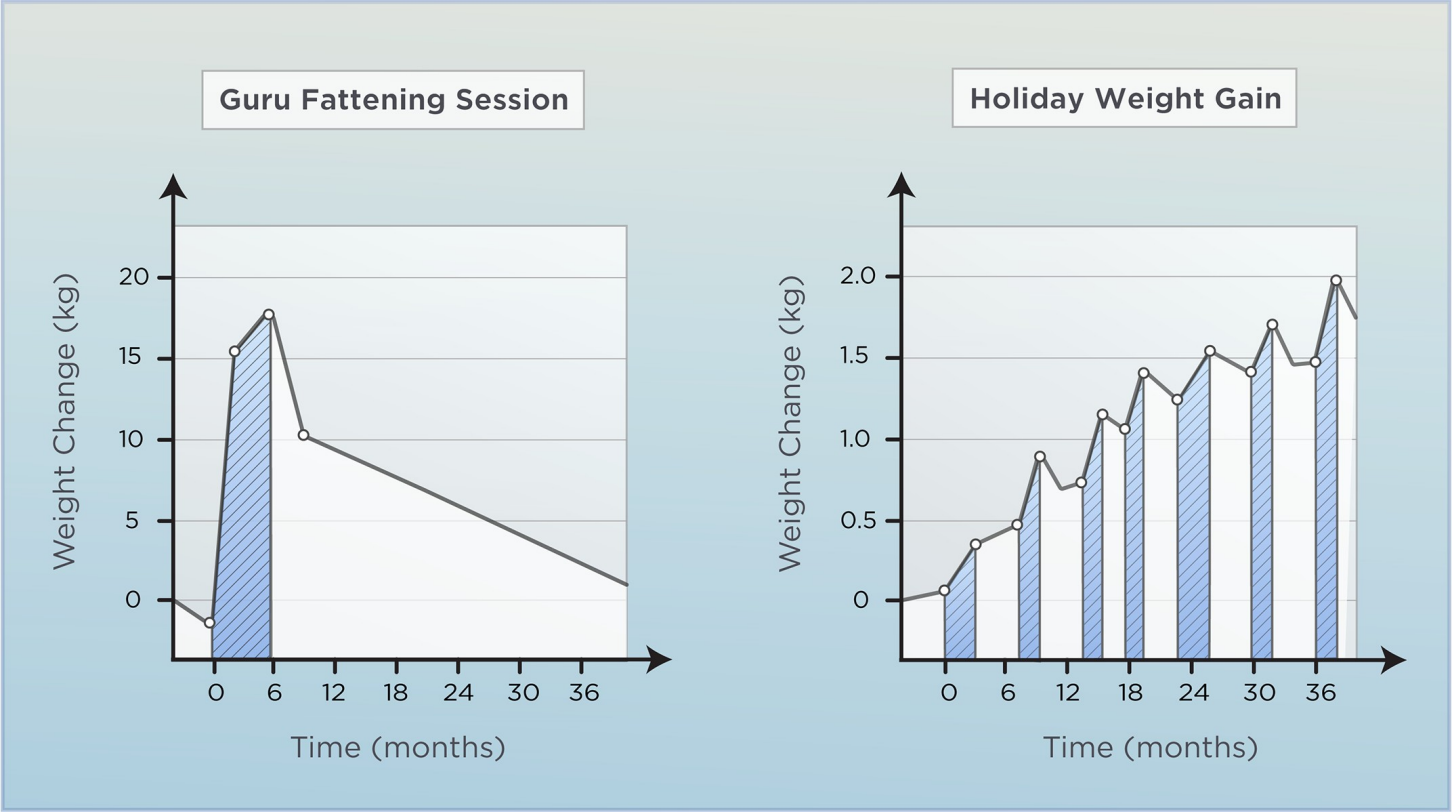
Naturally occurring periods of overfeeding: Lessons learned. Left: Body weight change over time due to a culturally determined overfeeding period, exemplified here by observations from the Massa of Northern Cameroon and their collective (Guru) fattening session amongst young males. Blue area highlights the overfeeding period. *Adapted after [45].* Right: Holiday weight gain showing the pulsatile change in body weight around holiday season in the 21st century. The slope of the curve suggests an incomplete weight recovery before the onset of the next holiday [49].

In some traditional cultures, young men and women participate in rituals of overeating as part of their pre-marriage preparation. In doing so, they acquire a degree of adiposity that is regarded as aesthetically attractive [42,43] (**Fig 3**). One observation made by anthropologists studying these cultures is the variability in individual weight gain. Although some individuals have no problems becoming overweight, others are effectively resistant to the ritual and have to work harder to increase their fat mass [42,44,45]. A somewhat similar observation can be made by looking at holiday weight gain, a phenomenon that occurs on a seasonal basis in high-income countries [46,47]. These overeating-promoting periods have been reported to give rise to a body weight gain of approximately 0.5 kg on average [48,49] (**Fig 3**), and just like the traditional rituals, holiday weight gain varies between individuals. Interestingly, it seems to affect primarily the part of the population that is already overweight [49], suggesting that a defective defense against overfeeding is involved in the etiology of obesity.

The weight gain variability observed in these natural experiments complements findings from human overfeeding interventions such as the infamous Vermont State Prison overfeeding study performed by Ethan Sims in the 1970s. In this classical study, only a few of the inmates were able to readily gain weight during 10 weeks of overfeeding, and most had a hard time forcing themselves into a fatter state [50,51]. Similar variance has been observed in other, less extreme overfeeding studies [52–54], and twin studies indicate that this is partly due to genetic factors [55,56]. In very distinct cases, this difference can be captured by the terms “easy” and “hard” gainers [53].

Following overfeeding, body weight tends to return to baseline. This homeostatic response is well-documented in rodents and has been observed in humans as well [33]. In one overfeeding experiment, pairs of monozygotic twins gained approximately 8 kg in response to a total energetic surplus of 84,000 kcal. After 4 months of free living, 7 of those 8 kg had been lost, and fat mass was largely normalized [57]. Interestingly, that study also indicated that genetic factors are involved in determining how humans recover from overfeeding [57]. Another study, however, showed that not all of the gained weight is lost following overfeeding [54]. This observation is supported by studies of holiday weight gain [48,49] (**Fig 3**) and might be explained by homeostatic inaccuracies, which lead to an insufficient lowering of food intake in most people following overfeeding [58,59]. Another explanation for the variation in response to overfeeding is the heterogeneous protocols used in the studies, including differences in the duration of overfeeding [60,61], total caloric surplus [58], and diet composition [62]. For example, an overfeeding study of short duration (3 days) did not lead to a decrease in food intake after the overfeeding [60], whereas a 21-day overfeeding protocol resulted in pronounced hypophagia once subjects returned to ab libitum conditions [61]. It is also important to highlight that the recovery of body weight following overfeeding not only may relate to the intake component of energy balance but also may implicate compensatory adaptations in energy expenditure [63–65]. Although discrepancies in study protocols may underlie some of the conflicting results, studies of experimental overfeeding generally support the existence of compensatory mechanisms with great interindividual variance and at least a partial return toward baseline body weight.

Based upon the research and theories presented here, it can be speculated that evolution has equipped humans with a physiological feedback system that is activated when prolonged overfeeding pushes fat mass beyond the upper biological boundary. A central aspect of this system might be a secreted circulating factor that works to counteract excessive expansions of body fat stores. As mentioned previously, parabiosis studies strongly indicate that a catabolic factor is present within the circulation of overfed rodents. If this hypothetical factor exists, it probably acts by suppressing food intake and possibly by inducing energy-dissipating processes, causing body weight to reenter the range in which it is not biologically regulated, i.e., the zone of biological indifference.

When leptin was found in 1994, it fulfilled several of the criteria for a humoral signal of the overfed state, indicating that the missing component in body weight control had finally been uncovered [66]. However, shortly after the discovery of leptin, it was shown that obese individuals often are hyperleptinemic and that administration of recombinant leptin has limited effects on appetite and body weight [32]. These findings have been interpreted as evidence of “leptin resistance” in the obese state—analogous to the notion of insulin resistance in type 2 diabetes. Fast forward 25 years after its discovery, leptin's role as a fat mass–lowering hormone remains enigmatic [32,33]. Furthermore, although the potential use of leptin as an antiobesity agent is still being scrutinized [67], intriguing insights presented next suggest that the hypophagic response to overfeeding is mediated by another, yet mysterious, molecule.

## Fractionations of fat: The unidentified anorexigenic agent from adipose tissue

In the last 2 decades, an increasing amount of literature has suggested that additional adipostatic hormones exist and that they participate in the biological defense against adiposity [23,32,33,68–70]. Whether such factors are dependent upon functional leptin signaling, as suggested by some [15,33,71], or whether they work in a leptin-independent manner [15,72,73] is unclear at the moment.

Around the time of the discovery of leptin, researchers showed that extracts of adipose tissue from overfed rats decreased food intake and body weight when injected into other rodents both peripherally and centrally [74,75]. By use of ultracentrifugation, Hulsey and Martin [74] separated an adipose extract into 3 molecular fractions: (1) >100 kDa, (2) 30‒100 kDa, and (3) 10‒30 kDa. Interestingly, the fraction containing molecules at a size of 30‒100 kDa was shown to decrease both food intake and body weight in response to daily intracerebroventricular injections for 7 days. The authors proposed that this specific fraction mediated the anorectic effect of the extract, and they termed the responsible agent “adipose satiety factor” [74]. Because leptin is only a 16-kDa protein, this finding implies that expanding white fat secretes other, currently uncharacterized, satiety signals. However, leptin has been reported to form complexes with other serum proteins, and it can therefore not be excluded that leptin was present in the 30‒100-kDa molecular fraction [76]. Conversely, another study reported that leptin cannot fully explain the satiating effect of adipose-conditioned media from overfed rats [71]. Hence, when leptin-deficient mice were administered such media, food intake decreased by 78%. An intraperitoneal bolus injection, however, of recombinant murine leptin at a dose equivalent to that found within the media did not affect feeding. In fact, it was shown that a 20-fold higher dose of leptin than that found within the media was needed to produce the level of hypophagia induced by adipose-conditioned media [71]. Moreover, rodents overfed by infusion of liquid diets through gastric tubing compensate for the weight gain by decreasing their voluntary intake of chow [15,17,77]. This response can be observed in both wild-type and genetically obese rodents [15–17]. In wild-type mice and rats, this adaptive hypophagia continues for several days postoverfeeding until body weight has normalized [15–17]. In contrast, rats with defective leptin receptors return to their inherent hyperphagic behavior within just 1 day after cessation of overfeeding. Despite this abnormal response, they still show a very subtle suppression of food intake in the postoverfeeding phase [15]. These findings suggest at least 2 things: (1) leptin signaling is required for engaging a proper and persistent hypophagic response to overfeeding, and (2) nonleptin signals exert a hypophagic effect on their own.

Another important point to highlight is the clear mismatch between plasma leptin and the quantity of calories consumed following an extended period of severe energy surplus. To fulfill the role as the sole hypophagic factor of overfeeding, plasma levels of leptin should remain elevated throughout the entire hypophagic phase and until fat mass has normalized. However, several studies show that this is not the case. Although the amount of circulating leptin increases profoundly during overfeeding, it rapidly returns to baseline just a couple of days after overfeeding has ended, while the animals are still hypophagic [15–17]. As White and colleagues have asked, "How can leptin suppress food intake when its levels are no longer elevated?" [15]. One possibility, however, is that temporal increases in leptin have long-lasting neuromodulatory effects on feeding circuitries [78,79]. Thus, although it is generally acknowledged that leptin contributes to postoverfeeding anorexia, the aforementioned findings merit a search for another factor with a pharmacokinetic profile that matches the time course of the hypophagic period.

## Through thick and thin: Parabiotic signals that make animals slim

Although the observations presented herein support the idea that weight gain is counteracted by additional fat-derived satiety factors acting alongside leptin, it is also important to emphasize that parabiosis experiments indicate the potential existence of 3 circulating signals that all contribute to lower fat mass, albeit by different mechanisms [66,68,70]. In 1959, Hervey carried out a parabiosis study in which one of the parabionts was exposed to hyperphagia-inducing lesions within the ventromedial hypothalamus. As a consequence, the lesioned rat became obese. Its parabiotic partner, however, lost interest in food and experienced a dramatic drop in body weight. It was suggested that these remarkable effects were caused by a humoral satiety signal that was secreted from the obese rat into its nonlesioned partner in which the signal subsequently suppressed food intake by acting upon the functional hypothalamus [80]. In 1984, Harris and coworkers used a similar parabiotic setup but made rats obese by tube-overfeeding instead of hypothalamic lesions [81]. In this study, parabiotic partners of overfed rats also lost large amounts of fat mass, but because they did not significantly decrease their food intake, the observed lipid-depleting effect was hypothesized to be mediated by an “antilipogenic factor” [70,81]. In more recent years, Harris has proposed the existence of a third signal that seemingly suppresses fat mass in a leptin-dependent manner [68,70,82]. Many intriguing questions surround these molecules, and it remains unclear whether leptin and the Hervey factor are the same signal [66,68].

## Searching the serum: From where does the unidentified factor of overfeeding originate?

**Fig 4:**
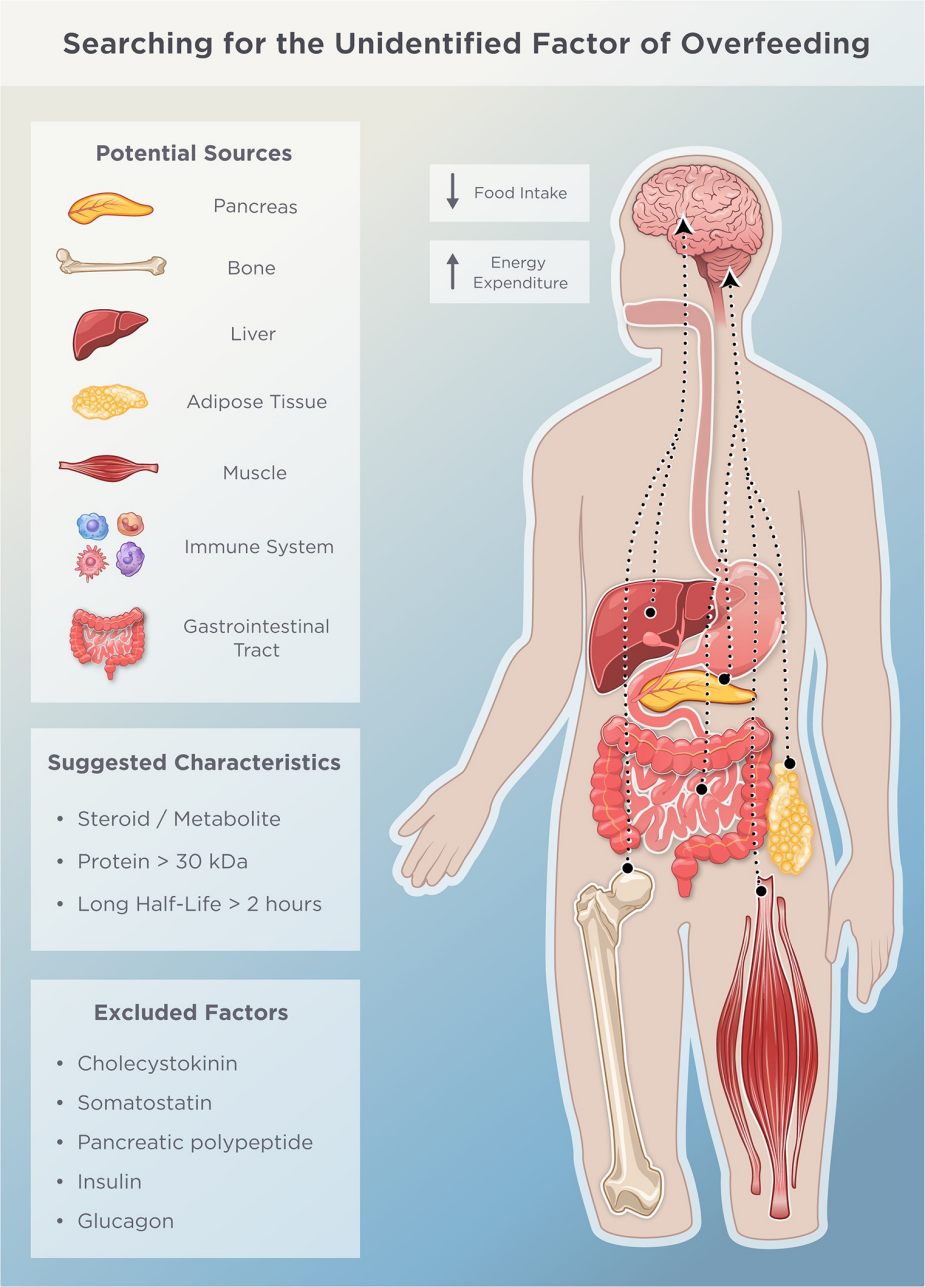
Possible origins of the unidentified factor of overfeeding. The putative circulating factor of overfeeding could be produced by a number of tissues including muscle, bone, pancreatic, hepatic, and adipose tissue. This blood-borne molecule is released from the tissue and works directly on appetite circuits within the central nervous system and/or indirectly by initially interacting with other peripheral organs. Its suggested chemical characteristics and previously excluded factors are mentioned in the box inserts.

Since the lipostatic theory was first proposed in the 1950s [21], it has been an attractive idea that expanding white fat secretes satiety factors into the circulation [22,33,69,83]. Although leptin serves to inform the brain about the amount of calories stored in fat depots [26], another line of thinking suggests that a secreted catabolic factor might reflect a functional aspect of adipose tissue [33]. According to this hypothesis, certain cell types might secrete such a molecule in response to intracellular lipid deposition, particularly when maximal storage capacity has been reached. Apart from adipocytes, other cell types are capable of taking up lipids. Immune cells residing in adipose tissue and also cells within skeletal muscle, liver, heart, and pancreas are exposed to ectopic fatty acids, which tend to end up in these organs upon overfeeding. Like adipocytes, these cells could be the source of a catabolic factor [33] (**Fig 4**), especially when considering that a series of inflammatory signals associated with obesity have been linked to altered food intake [84]. This idea that an “unidentified factor of overfeeding” could originate from other cells than adipocytes are in line with studies suggesting that overfeeding leads to the release of fat mass–lowering signals of either hepatic, pancreatic, or gastrointestinal origin [83] (**Fig 4**). Although it is largely speculative whether the liver and the gastrointestinal tract secrete such substances, there is actual evidence to support the possibility that pancreatic islets play a key role in the defense against adiposity [69]. As such, transplantation of islets from normal mice into genetically obese (leptin-deficient) mice has been reported to pause normal weight gain, and upon removal of the islets, mice start to regain weight [85]. Apart from these classical metabolic organs, it is also noteworthy that weight-bearing bones have been suggested to participate in body weight regulation [86], purportedly via a leptin-independent satiety signal that is secreted from osteocytes in response to loading stress [73] (**Fig 4**).

## Unidentified hormonal protection against weight gain: A look to the future

Since Hervey first provided experimental evidence for the existence of a blood-borne anorectic agent, more than a dozen parabiosis studies have been published [23], and a wealth of knowledge has been acquired about the complex neuroendocrine regulation of food intake [3,7]. Yet, one of the most important aspects of body weight regulation remains an unsolved mystery: What are the biological mechanisms that defend against adiposity? Although afferent signals undoubtedly play a role in metabolic feedback, mounting evidence underscores that protection against weight gain involves unidentified blood-borne factors that act in either a leptin-dependent or a leptin-independent manner. Unlike the previous generations of obesity biologists, we are no longer limited by traditional biochemical instruments. With the newest technological advances, including omics-methods, the time is ripe for pursuing unknown circulating factors and for delineating their mechanisms of action. The future is now, and the unidentified factor of overfeeding, if this signal exists, is there to be discovered. Decoding the physiology that counteracts weight gain is arguably one of the most critical tasks for modern metabolic research, and we hereby encourage our colleagues to join this 60-year-old quest.
